# Supplementary material for: Multiple Copies of microRNA Binding Sites in Long 3′UTR Variants Regulate Axonal Translation
Source: Cells. 2023 Jan 6;12(2):233. doi: 10.3390/cells12020233 (PMC9856650; doi:10.3390/cells12020233)
Supplement: Supplementary file 1 [file cells-12-00233-s001.zip › cells-1889740-supplementary.pdf]

# - Supplementary figures -

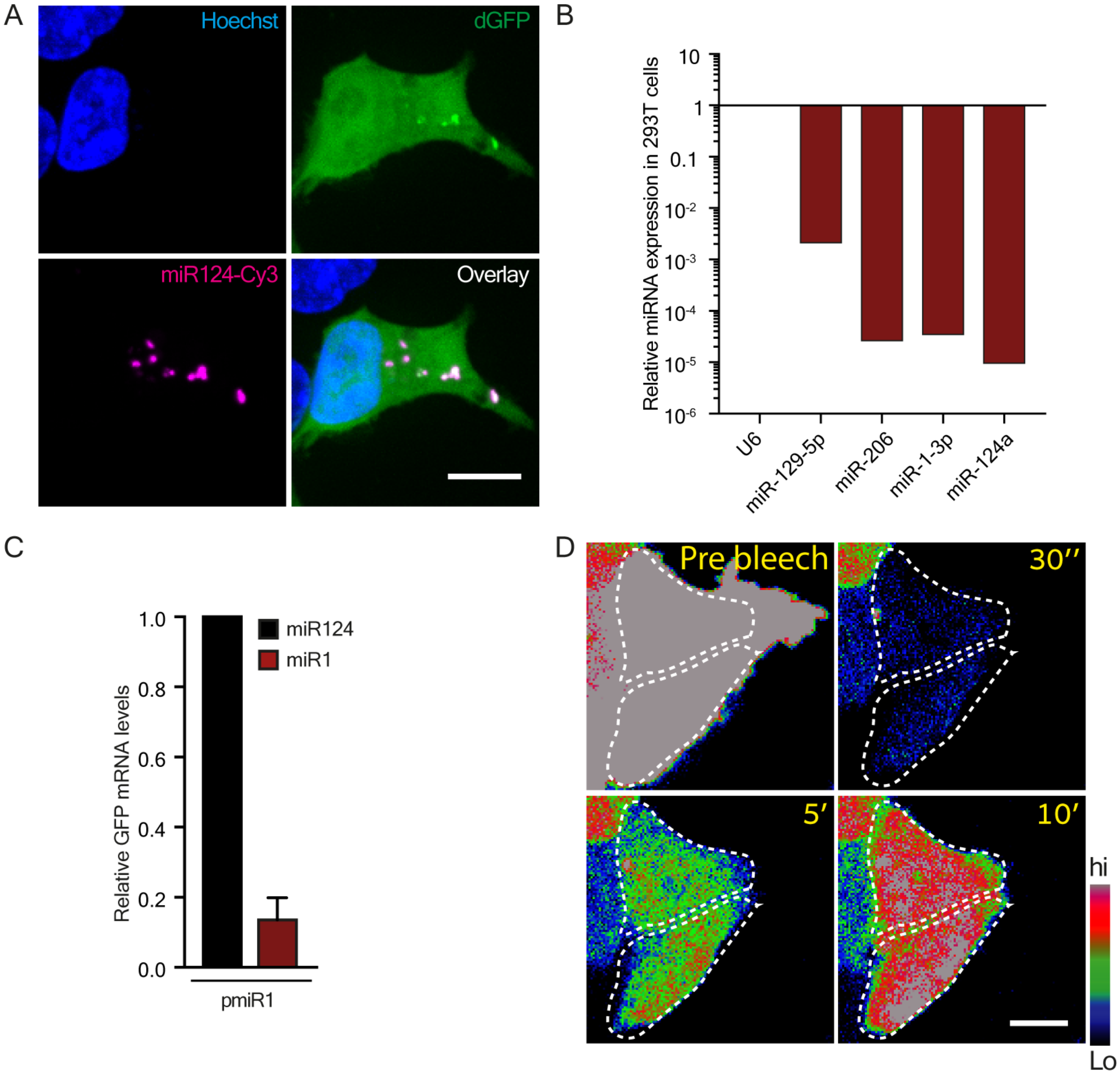

Supplementary Figure S1 – **Experimental system setup. A)** A representative image of an HEK-293T cell co-transfected with dGFP (green) and with mature Cy3-miR-124-3p (magenta). Cell nucleus was labeled with Hoechst dye (blue). Scale bar = 10µm. **B)** RT-qPCR analysis of the relative miRNA expression in 293T cells. miRNA relative expression was normalized to U6 expression (1). The Y axis is in log scale. **C)** RT-qPCR analysis of relative WT 3'UTR GFP (full complementation) mRNA levels at 24 hours after transfection with miR-1-3p, or with non-specific miR-124a-3p. **D)** Representative images of dGFP FRAP experiments. Images show dGFP signal prior photobleaching and 30 seconds, 5 minutes as well as 10 minutes after photobleaching in HEK293T.

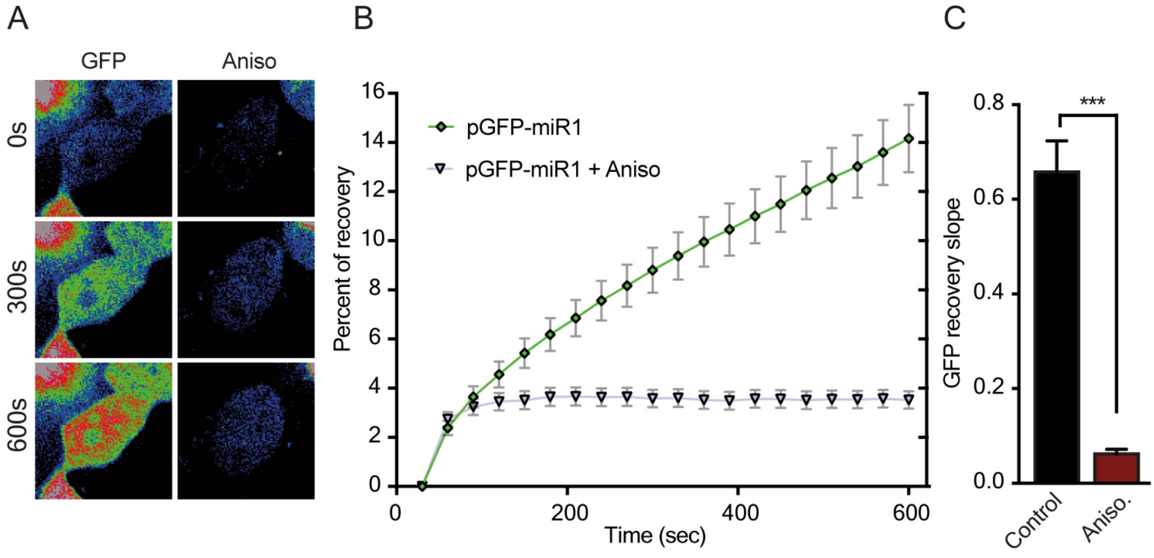

Supplementary Figure S2 – **Validation of dGFP signal buildup assay as a reporter for protein synthesis. A)** Representative images of dGFP in 293T. The images show three time points for each cell: 0 minutes, 5 minutes, and 10 minutes after photobleaching in control cultures, or cultures treated with 40µM Anisomycin. **B)** A graph showing the cumulative percent of fluorescence of dGFP during the 10 minutes following photobleaching. **C)** Quantification of the mean slope of dGFP recovery after photobleaching. *** p-value < 0.001.

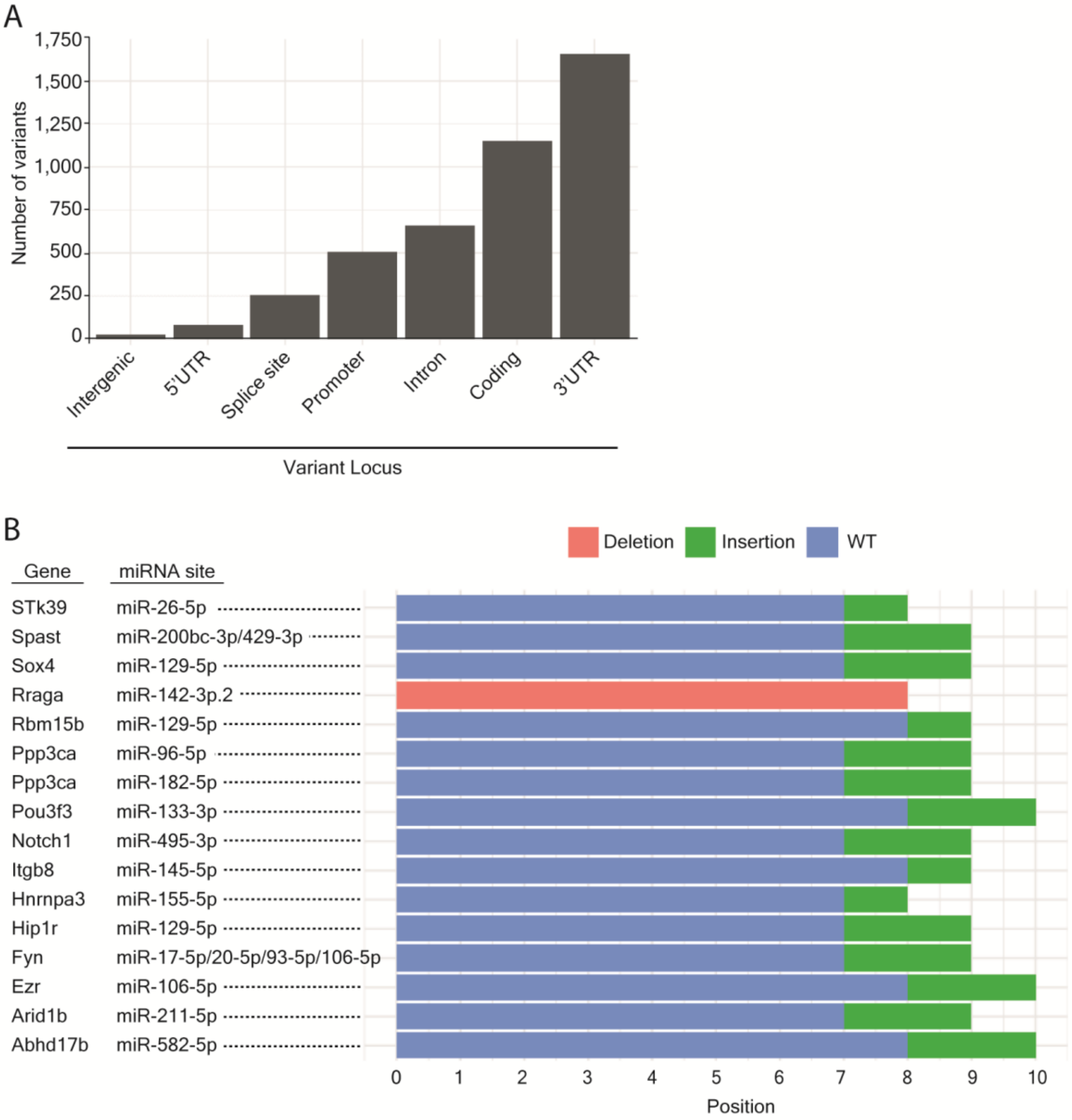

Supplementary Figure S3 – **Variant-calling analysis reveals that 3'UTR variations in miRNA seed regions of axonal mRNAs are extremely rare. A)** Segmentation of the number of variants in axons versus soma by the site of variation within the mRNA sequence. **B)** A graph presenting all identified mRNA variants with modified seed regions in axonal transcriptome, and specification of the positions modified.

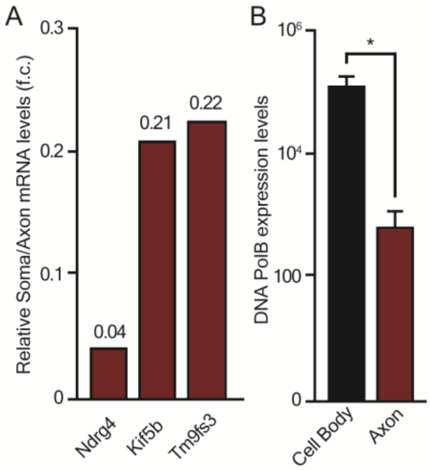

Supplementary Figure S4 – **Validations of axonal purity and NGS data.**

A) NGS differential expression results for mRNA with significantly longer 3'UTR: Ndrg4, Kif5b, and Tm9fs3 in axons compared to cell bodies. **B)** Relative mRNA levels of DNA polymerase II (PolB) in cell bodies vs. axonal RNA. The Y axis is in log scale. Two-tailed students t-test. *p-value < 0.05.

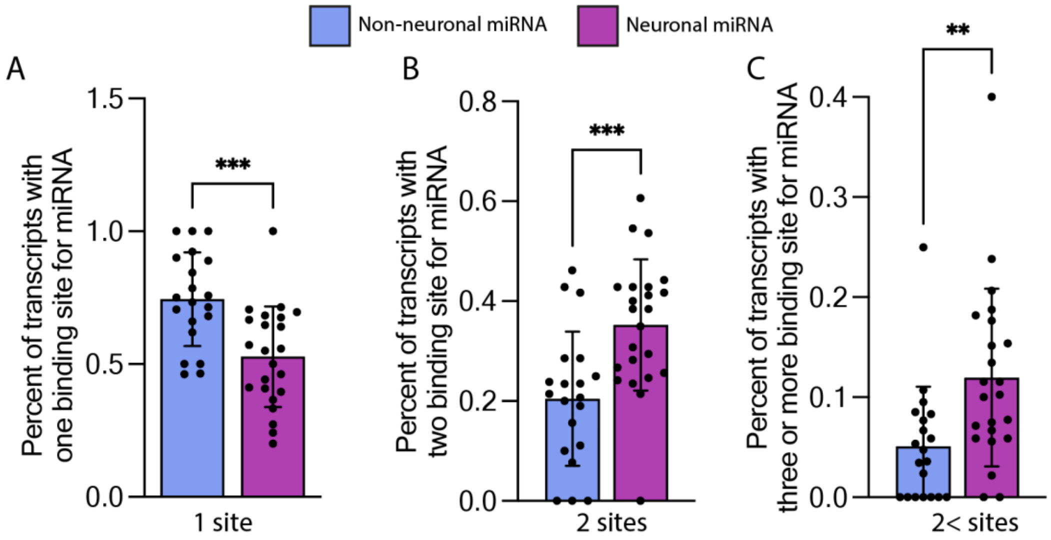

Supplementary Figure S5 – **Quantification of the miRNA target sequences in long-3'UTR axonal variants.** The mouse targets data bases for 20 non-neuronal and 22-neuronal miRNA was acquired from Targetscan.org and were aligned with the most significant axonal transcripts with long 3'UTR (figure 4B). For each miRNA, the ratio of transcripts containing one, two, or more than two binding sites was calculated. Two-tailed, unpaired students' t-test. ** $p<0.01$, *** $p<0.001$.

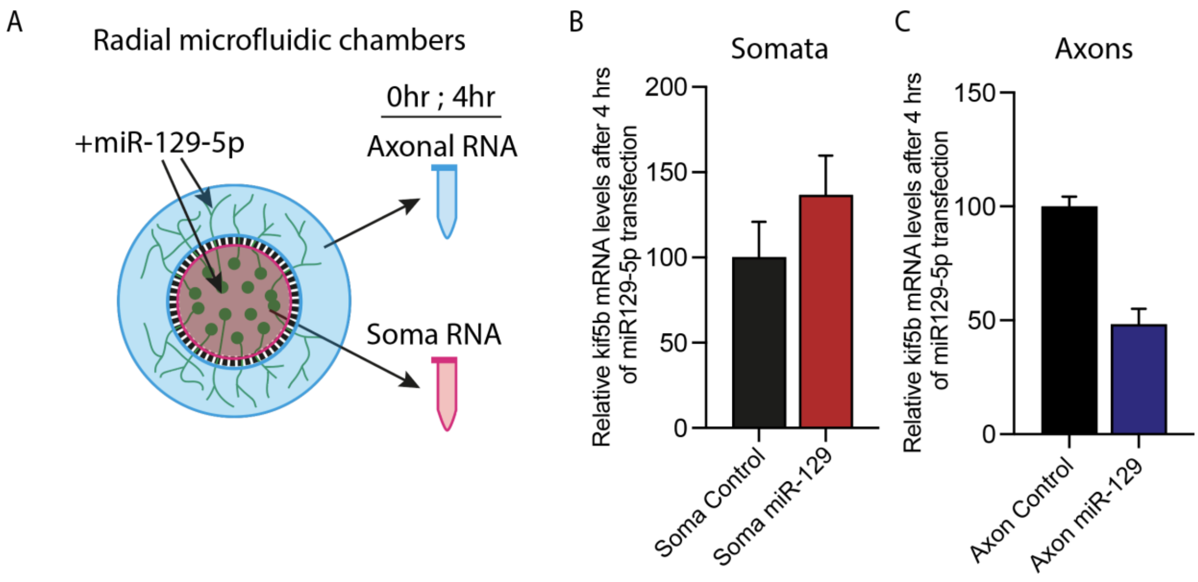

Supplementary figure S6 – **Axonal kif5b transcripts are prone to rapid decay by miR-129-5p.** **A)** Primary motor neurons were cultured in the central compartment (red) of radial microfluidic chambers. At 14DIV, after axons have massively crossed into the outer compartment (blue), axons and cell bodies were both transfected with either miR-129-5p mimic or by mock transfection. RNA was harvested separately from axonal compartment and soma compartment immediately after transfection, or 4-hours after transfection. **B-C)** qPCR analysis of relative mRNA levels at 4 hours post-transfection compared to time-0 in somta **(B)**, and in axons **(C)**.
